# Habitat Composition and Connectivity Predicts Bat Presence and Activity at Foraging Sites in a Large UK Conurbation

**DOI:** 10.1371/journal.pone.0033300

**Published:** 2012-03-12

**Authors:** James D. Hale, Alison J. Fairbrass, Tom J. Matthews, Jon P. Sadler

**Affiliations:** 1 School of Geography, Earth and Environmental Sciences, The University of Birmingham, Birmingham, United Kingdom; 2 Imperial College London, Silwood Park Campus, Ascot, United Kingdom; 3 School of Geography and the Environment, University of Oxford, Oxford, United Kingdom; University of Western Ontario, Canada

## Abstract

**Background:**

Urbanization is characterized by high levels of sealed land-cover, and small, geometrically complex, fragmented land-use patches. The extent and density of urbanized land-use is increasing, with implications for habitat quality, connectivity and city ecology. Little is known about densification thresholds for urban ecosystem function, and the response of mammals, nocturnal and cryptic taxa are poorly studied in this respect. Bats (Chiroptera) are sensitive to changing urban form at a species, guild and community level, so are ideal model organisms for analyses of this nature.

**Methodology/Principal Findings:**

We surveyed bats around urban ponds in the West Midlands conurbation, United Kingdom (UK). Sites were stratified between five urban land classes, representing a gradient of built land-cover at the 1 km^2^ scale. Models for bat presence and activity were developed using land-cover and land-use data from multiple radii around each pond. Structural connectivity of tree networks was used as an indicator of the functional connectivity between habitats. All species were sensitive to measures of urban density. Some were also sensitive to landscape composition and structural connectivity at different spatial scales. These results represent new findings for an urban area. The activity of *Pipistrellus pipistrellus* (Schreber 1774) exhibited a non-linear relationship with the area of built land-cover, being much reduced beyond the threshold of ∼60% built surface. The presence of tree networks appears to mitigate the negative effects of urbanization for this species.

**Conclusions/Significance:**

Our results suggest that increasing urban density negatively impacts the study species. This has implications for infill development policy, built density targets and the compact city debate. Bats were also sensitive to the composition and structure of the urban form at a range of spatial scales, with implications for land-use planning and management. Protecting and establishing tree networks may improve the resilience of some bat populations to urban densification.

## Introduction

Fifty years of agricultural intensification, fragmentation and urbanization have radically altered the landscape composition of the UK [1]. Urban areas have grown substantially over the last 20 years [2] and now support the majority of the global population [3]. Urbanization is characterized by an increase in sealed land-cover density [4], geometric complexity, fragmentation of land-use patches [5], [6] and a reduction in patch size [6], [7]. Combined with varying disturbance levels [8], this results in a spatio-temporally complex land-use mosaic [9], [10] with far-reaching consequences for species dispersal [11], ecological function [12] and ecological service provision [13]. In some countries, urbanization has resulted in urban sprawl into agricultural land [14], while in others policies favour compact city forms [15]. Where greenbelts constrain urban sprawl [16], there is evidence that urban landscapes have ‘densified’ and lost greenspace, especially over the last decade [17]. Little data exist that indicate how much densification the urban ecosystem can withstand before ecosystem function is substantially impaired. In terrestrial habitats increased urbanization generally has a negative effect on species richness, although this pattern is not universal [18]. Organism responses to increasing urban land-cover are species and trait-specific, but generally differentiate between generalist species that thrive or show humped abundance patterns, and specialist species that exhibit declines [8], [19], [20], [21], [22], [23].

Urban density thresholds for species presence and abundance are likely to be contingent upon sampling methodology and the spatial scale at which built density and landscape composition are measured. There is currently a multiplicity of approaches evident in the literature [24], [25], clarification is needed in order to improve comparability between studies and to aid the translation of results into conservation practice. Clarity may be gained by studying taxa whose species are sensitive to different measures of urbanization at a range of spatial scales, as well as to the surrounding landscape. There is already a considerable literature on birds and urbanization [20], [22], [25], but their life histories and responses to urbanization do not always reflect those of other groups [26]. Mammals, nocturnal and cryptic taxa are poorly studied in this respect and bat (Chiroptera) communities are ideal candidates for research. They typically include species that exploit built structures [27] are sensitive to landscape scale, patch effects [28], [29] and to changes in structural connectivity [30], [31].

The few studies focusing on the effect of urbanization on bat species indicate variability in response to changing urban form at a species, guild and community level. In studies of cities in the Czech Republic [32], Mexico [33] and Australia [34] bat activity was lower in high density residential areas, than in low density areas (e.g. suburban, urban fringe) and semi-natural areas. In addition, lower species richness was reported in the urban centre and densely developed areas. This contrasts with other studies in the USA [28], [29] where positive relationships have been reported between both overall bat activity and species richness of natural habitat fragments and the urban density of the surrounding landscape. Several studies have identified positive relationships between urbanization and the activity of certain species [28], [29], [33], [35], but other species clearly favoured semi-natural areas or exhibited a broad tolerance of urbanization [32], [33]. It has been suggested that these responses reflect differences in wing and call morphology, with species specialising in cluttered habitats avoiding brightly lit and poorly vegetated urban areas [33].

Given the intensity of compositional change in urban areas [2], [17] and the associated high levels of fragmentation [5], one might expect that connectivity and linkage would be a central theme in urban ecology, as it is in other landscapes [1], [36], [37], [38]. Indeed, connective features such as green networks and corridors have been influential in guiding city planning in many areas of the world [39], [40], and the creation and preservation of wooded corridors does seem to present an ideal opportunity for restoration aimed at enhancing spatial population resilience in cities [41]. However, there are very few studies that focus on this element. Studies on plants and invertebrates [42], [43] in UK greenways identified multiple structural and functional roles that were species specific in terms of habitat provision, but did not indicate a strong functional conduit role that enhanced movement and dispersal. Although evidence that wooded linear features such as streets and riparian corridors facilitate connectivity for birds in urban areas [44], [45] there are few studies pertaining to urban bats, although several have identified relationships between linear features and bat activity in agricultural areas [30], [46], [47]. Such features appear to have roles in both feeding and movement and thresholds for loss of functional connectivity are still unclear [35], [48].

Here we explore the influence of urban landscape composition and structural connectivity on the presence and activity of bats at a range of spatial scales. We stratified sampling sites evenly across classes of urban form whose composition and extent were clearly defined *a priori* using a wide range of environmental data captured in a Geographical Information System (GIS). Foraging sites with similar local land-cover were selected to reduce the effect of confounding local variation in habitat type, and their landscape context measured consistently at multiple spatial scales. A proxy measure of functional connectivity was developed for each scale based on the traits of the species encountered. Both walking surveys and fixed position detectors were used to record bat activity. Using the assemblage and environmental data we addressed the following research objectives: (1) To characterize bat activity and presence in relation to urban density and landscape composition; (2) To assess the spatial scale at which species respond to the urban landscape; (3) To establish the significance of connectivity for bat activity in a heavily urbanized landscape.

We achieved our objectives and explore in the Discussion section the issues surrounding quantifying land-cover, land-use, functional connectivity and species specific responses to landscape change. Despite some progress with mapping key variables at a high spatial resolution and large spatial extent, our proxies for roost potential (large trees and residential buildings) and lighting (road area) did not add any explanatory power to the models and could be improved on. Future studies may benefit from data on tree species, building age and densities of lighting columns.

## Results

**Figure 1:**
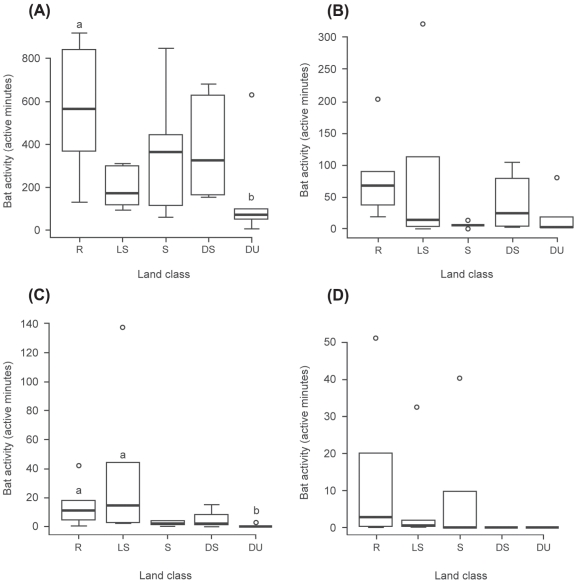
Bat activity adjacent to survey ponds based on full-night survey data. Land classes follow an urbanization gradient from Rural (R) to Dense Urban (DU). Box plots represent total active minutes for (A) *P. pipistrellus*, (B) *P. pygmaeus*, (C) a group comprising *N. noctula*, *E. serotinus*, *N. leisleri* and (D) *M. daubentonii*. Boxes that do not share a letter showed significant differences (P<0.05) between land classes.

We recorded bat calls within a total of 14,176 survey minutes using the fixed-point automatic detector. Of these, 11,545 minutes contained calls identifiable to species or guild level. These included 9,950 active minutes (86% of the identifiable bat calls) of *P. pipistrellus* calls, 1,330 (11%) of *P. pygmaeus* and 345 (3%) belonging to the NSL (*Nyctalus noctula*, *Eptesicus serotinus*, *Nyctalus leisleri*) guild, with some minutes including calls from more than one species/guild. Walking surveys added an additional 1178 active minutes (75%) for *P. pipistrellus*, 190 (12%) for *P. pygmaeus*, 49 (3%) for the NSL guild and 163 (10%) *Myotis* calls, all of which where from bats observed feeding over the pond surface and were therefore confirmed as *Myotis daubentonii*. Bats were recorded at all of the thirty survey sites. Only *P. pipistrellus* was recorded at all sites, although *P. pygmaeus* was recorded at 93.3% of sites. The NSL guild was recorded at 73.3% of sites but was virtually absent from those within the Dense Urban land class (Fig. 1). *M. daubentonii* was present at 33.3% of sites and was negatively associated with increased urbanization (Fig. 1). Full-night activity for both *P. pipistrellus* (p = 0.043) and the NSL guild (p = 0.035) was significantly higher in the Rural compared to the Dense Urban land class (Fig. 1). Evening activity for *P. pipistrellus* and the NSL guild followed similar patterns, although the differences between classes were not significant.

**Figure 2:**
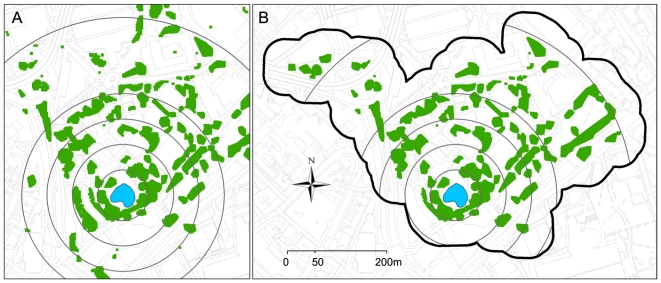
A survey pond and two methods used to extract landscape data at a multiple scales. (A) An unrestricted extraction of landscape data using concentric buffers. (B) Connected (available) landscape mask created using tree networks buffered by 50 m. This polygon was used as a mask to restrict the landscape analysis to the area within this network. In both examples, landscape data were extracted at distances of 50, 100, 150, 200, 350 and 500 m from the pond centre.

**Figure 3:**
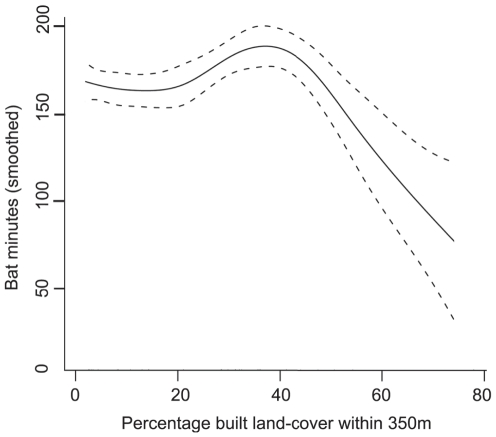
Partial plot of smoothed evening bat activity and percentage built land-cover within 350 m of surveys sites. This was included in the final model for evening activity of *P. pipistrellus* (see Table 1 for full model).

**Figure 4:**
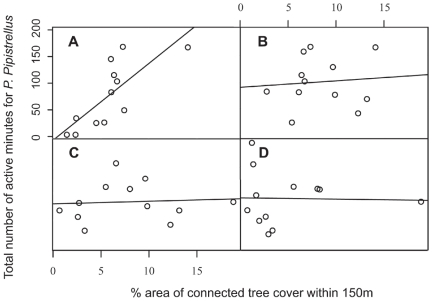
Conditional scatter plot of *P. pipistrellus* evening activity against connected tree cover (>6 m) within 150 m. (A) sites within Dense Urban and Dense Suburban (B) Dense Suburban and Suburban (C) Suburban and Light Suburban, (D) Suburban and Rural land classes.

Multiple models were created for all species and guilds and the best of these are presented in Table 1. These models included data extracted using concentric buffers applied both to the landscape around each pond (concentric landscape) and restricted to the landscape intersected by a connectivity mask (connected landscape) (Fig. 2). In general, *P. pipistrellus* activity was highest at sites surrounded by low or moderate levels of built land-cover or at well-connected sites in highly urban areas. The best models all included the area of built land-cover within 350 m of survey ponds, with activity peaking at intermediate levels of built land-cover and being lower but more variable at high levels of urbanization (Fig. 3). These models included a positive association with connected tree cover (>6 m high) within a radius of 150 m for sites in Dense Urban and Dense Suburban land classes (Fig. 4). Evening activity was also positively associated with connected garden area within both 50 and 500 m. These garden parameters were not present in the full-night model, but were replaced by a measure of connected vegetation cover within 200 m of the site (Table 1).

**Table 1:** Summary of the best-fit multi-scalar models for measures of bat activity and presence.

Response	Duration	Model type	Landscape variables and spatial scales (radii in m)								AIC
			50	100	150	200	350	500	750	1000	
PP	Evening	GAM Ne	Gard***		Tree>6 m***s(Urb)		Bu***s‡	Gard*			283.6
PPy	Evening	GLM Ne		-Tree>6 m**						Ve**	187.8
My	Evening	GLM Bi				Nat** ‡					32.2
My	Evening	GLM Bi						Nat** ‡			32.8
My	Evening	GLM Bi			Nat** ‡						33
NSL	Evening	GLM Bi						-Bu** ‡			35.2
NSL	Evening	GLM Bi							-Bu** ‡		35.2
NSL	Evening	GLM Bi								-Bu** ‡	35.3
PP	Full-Night	GAM Ne			Tr>6 m***s(urb)	Ve*	Bu***s‡				364
PPy	Full-Night	GLM Ne			-Tr>6 m**					-Bu* ‡	270.3
PPy	Full-Night	GLM Ne				-Tr>6 m**				-Bu* ‡	270.7

‡ Indicates data extracted using simple concentric circular buffers, otherwise, a connectivity mask (a 50 m buffer around tree networks) was used.

* = P<0.05,

** = P<0.01,

*** = P<0.001.

The most parsimonious model for each response variable is shown, according to Akaike Information Criterion (AIC) values. PP indicates *P. pipistrellus*, PPy *P. pygmaeus*, NSL a group comprising *N. noctula*, *E. serotinus*, *N. leisleri* and My indicating *M. daubentonii*. Models whose AIC value≤2 of the optimum model are also included. Model type is either a generalised linear model (GLM) or a generalised additive model (GAM) with either a negative binomial (Ne) distribution for activity data or binomial (Bi) for presence. See Table 2 for variable definitions.

*Pipistrellus pygmaeus* activity was highest at ponds located within a highly vegetated landscape but poorly connected at a local level. All models included negative parameter coefficients for connected tree cover (>6 m high) within a radius of 100–200 m. Evening models included a positive relationship between activity and connected vegetation within 1 km, and the full-night models included a negative association with the area of built land-cover within 1 km.

No valid GLM or GAM activity models were identified for *M. daubentonii*, or the NSL guild, but several logistic regression models were selected for evening presence data. The evening presence model for *M. daubentonii* included a positive relationship with natural land-cover (concentric) within 150 to 350 m of the survey sites (Table 1). For the NSL guild evening activity was negatively associated with built land-cover (concentric) within 350–750 m (Table 1). For these species, restricting the landscape analysis to the areas adjacent to tree networks (connected landscape) did not result in any valid models. Candidate all-night NLS presence-absence models all exhibited residual spatial patterning (e.g. Figure S1). We used a spatial correlation structure to compensate for this, but it did not improve either the residual spread or the AIC of the models, so all the models were rejected.

## Discussion

**Figure 5:**
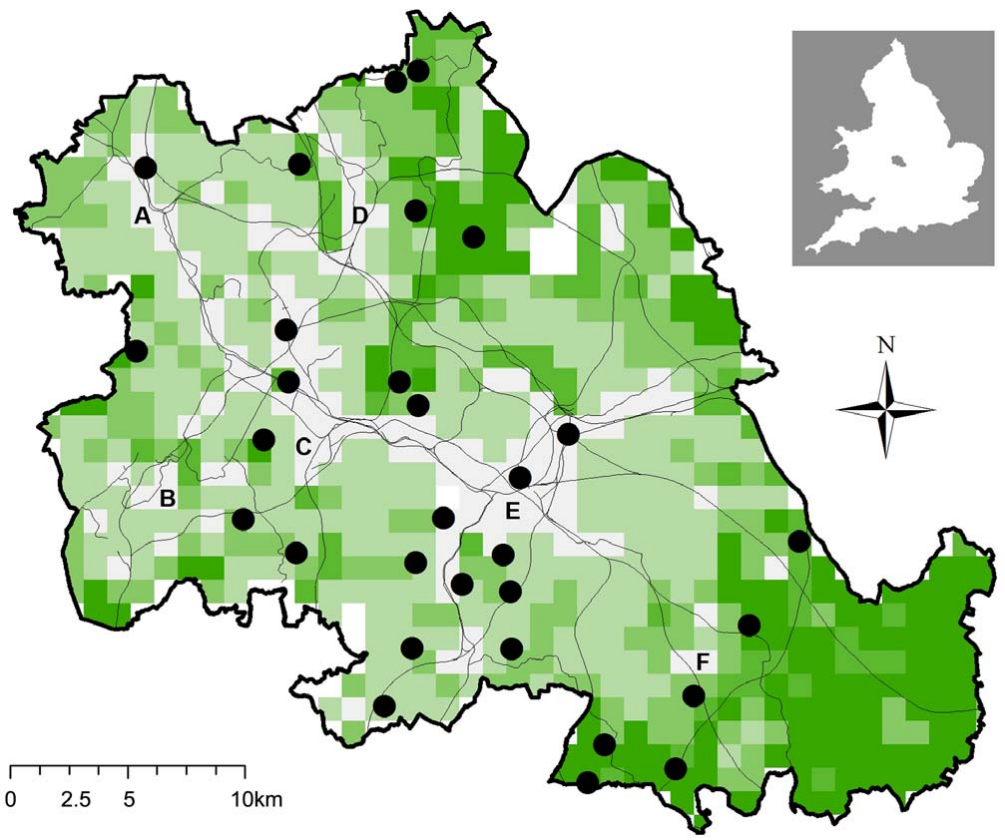
The West Midlands metropolitan county study area. It includes the metropolitan borough centres of A Wolverhampton, B Dudley, C Sandwell, D Walsall, E Birmingham and F Solihull. Bat survey ponds are indicated by a black circle and were stratified by urban land classes, which are represented by a grid of 902×1 km^2^pixels covering the study area. These range from Dense Urban (white) to Rural (dark green). Canals and railways are indicated by fine black lines.

**Figure 6:**
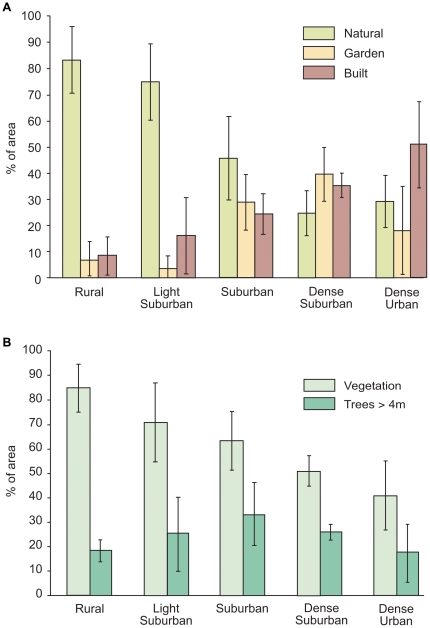
Landscape data summaries for 1 km^2^ circles surrounding each pond. (A) Mean area and SD for 3 of the 13 land-cover and land-use types derived from the Ordinance Survey Mastermap and used to assign ponds to land classes (see Table S1). (B) Mean area and standard deviation for vegetation cover and trees>4 m high.

We investigated the response of a bat community to urbanization, landscape composition and structural connectivity at a variety of spatial scales using standardized samples across five urban landscape classes (Figs. 5 & 6), targeting small ponds with consistent levels of adjacent riparian woodlands. All species were found to be sensitive to at least one measure of urbanization and some were additionally influenced by landscape composition and structural connectivity at different spatial scales. For two species, habitat associations differed between evening and full-night models.

### Urban Density

Three broad measures of urbanization were derived for this study (i) the area of built (sealed) land cover parcels derived from OS digital data, (ii) the area of natural (vegetated) land-cover parcels and (iii) the area of vegetated (remotely sensed) land-cover (Table 2). It is notable that all of the species/guilds in our study were found to be sensitive to at least one of these measures of urban density, given the limited evidence on the response of bat communities to urbanization [28], [48]. Each measure provided significant explanatory power to different models, supporting calls for the use of multiple measures of urbanization in gradient studies [24]. It is likely that these measures differ in their representation of key resources or ecological disruptors as they are broad and indirect measures of a complex anthropogenic gradient [25]. Although most species demonstrated a negative association with urbanization, we found a non-linear relationship between *P. pipistrellus* activity and built surface cover. Activity peaked at ∼40% built cover, yet at levels above ∼60% activity rapidly reduced, implying the existence of a threshold or tipping point [49]. This is the first report of such a relationship for a bat species, although non-linear relationships with urbanization have been identified for other taxa [20]. *Pipistrellus pipistrellus* could be described as an “urban adapter” [50], whilst the remaining species would be “urban avoiders” of varying sensitivity. These results broadly agree with those of other studies, for example, that *Myotis* species tend to avoid villages [51] and urban centres [32]. European work [32] suggests that small bats with low wing loadings are tolerant of even dense urbanisation, but large bats with high wing loadings generally avoid urban centres. This is, however, at odds with work in Australia which suggests the reverse [52] and also in disagreement with studies of urban bird traits, which suggest large size/wings are a trait of urban adapters [21]. These differences may be an artefact of differences in urban composition and morphology between European and Australian cities, the scale that urbanisation is measured at, how urban density is defined and the degree to which different species are willing to accept human subsidised resources (e.g. building roosts).

**Table 2:** Land-cover and land-use explanatory variables used in the analysis.

Variable	Abbreviation	Description
Water	Wat	Surface water features from OSM including canals, ponds and streams
Natural	Nat	Polygons dominated by vegetated/unsealed land-cover from OSM including roadside grass verges and parks but excluding gardens
Garden	Gard	Gardens as defined from the Ordnance Survey Mastermap (OSM) layer in 2008
Roads	Rds	Roads from OSM
Buildings	Build	Built structures from OSM
Built	Bu	Polygons dominated by built land-cover types from OSM including roads, buildings and pavements but excluding gardens
Vegetation	Ve	All vegetation cover at 2 m pixel resolution, extracted from aerial near-infrared and colour photography 2007 (Bluesky International Limited, Leicestershire
Urban density	Urb	Nominal variable (1–2) differentiating between highly urban (Dense Urban and Dense Suburban) and less urban land classes (Suburban, Light Suburban and Rural
Trees	Tree	Five tree datasets created by selecting areas of the (above) vegetation dataset ≥ a specified height* above the ground, according to photogrammetrically derived data collected in 2007 (Bluesky International Limited, Leicestershire, UK)
Connected area	ConA	Area of connected tree cover >4 m in height buffered by 50 m and intersecting each survey pond. Connectivity is defined here as a spatial network of tree patches separated by a maximum of 40 m
Edge	Edge	Length of the perimeter of the connected tree cover described above

Each was measured as the total area (m^2^) or length (m) within a radius of 50, 100, 150, 200, 350, 500, 750, 1000, 1250, 1500, 1750, 2000, 3000 and 4000 m

* Five datasets representing tree cover with a minimum height of 4, 6, 10, 15 or 20 m.

### Landscape composition

The often contradicting findings from studies of urban bat communities illustrate some of the broader challenges associated with attempting to identify ecological patterns along urbanization gradients [25]. The descriptions of the urban form provided by Gaisler et al. [32], Avila-Flores and Fenton [33] and Gehrt and Chelsvig [28] varied considerably in their detail. We addressed this issue by accessing high-resolution parcel based and remotely sensed data for land-cover and land-use for the entire study area.

Although all species demonstrated a negative response to broad measures of urbanization, considerable differences in activity were evident between sites at similar points along these gradients. This suggests that more subtle variations in landscape composition may also be important. *Pipistrellus pipistrellus* evening activity was found to be positively associated with gardens, which might be expected given their propensity for roosting in buildings [53] and for early evening emergence. An additional explanation is that gardens might typically provide tree cover that facilitates early emergence and feeding. These findings pose further questions about the mechanisms behind associations between bat activity and residential land-covers reported elsewhere [28], [29], [32], [54]. As with other studies, differences were found between the landscape composition preferred by *P. pipistrellus* and *P. pygmeaus*. A positive association between *P. pipistrellus* and local tree cover was expected given their known use of edges as commuting and feeding areas [30], [31] and their role in increasing the attractiveness of adjacent roost sites [55]. The association of *P. pygmeaus* with aquatic habitats described elsewhere [51], [56], [57] was not observed in this study. However, this may reflect variations in the quality of riparian vegetation [58], which we were unable to measure at a landscape scale.

We expected *M. daubentonii* to demonstrate a strong positive association with the area of water in the vicinity of the survey sites [59], yet we did not find support for this in our results. It is possible that by surveying ponds we removed water as a limiting variable or that the social dynamics of this species served to mask important habitat associations [60]. Members of the NSL guild are reported to seek out pastures, parks and other open green spaces [51]. Their relatively high wing loadings, medium-high aspect ratios and low call frequencies permitting them to hawk in the open, typically feeding on large insects [27]. Our presence models broadly support this, although we were unable to differentiate between ground vegetation types and management for the extent of our study area.

In general, we expected bat presence and activity to reflect the local availability of roosts [55] and foraging sites [51]. Several of our landscape variables were intended to be proxy measures for these key resources, yet the value of our roost metrics was limited. There is an inevitable trade-off between the detail and availability of urban habitat data and the spatial scale of analysis [25] and it is likely that greater effort is required to map roost potential effectively. In addition, both roosting bats [55], commuting bats [31] and their insect prey are sensitive to variations in microclimate, which in urban areas will be heavily influenced by human activity. Additional data that improves the integration of human processes such as land management and intensity of use into urban ecological models may therefore clarify how roosting or feeding potential varies within each land-cover type.

### Landscape connectivity

Our approach employed proxy measures of functional connectivity to estimate the areas of the landscape theoretically available to bat species that commute along tree networks and is an extension of the accessible habitat model [61], [62]. Functional connectivity is concerned with the ability of individuals to move between resource patches within the landscape rather than explicitly measuring the structure of landscape elements, although structure is frequently used as a proxy for function [63], [64]. The measures of structural landscape connectivity used to extract landscape variables from the GIS appeared to be a good approximation of functional connectivity for the two *Pipistrellus* species. Euclidian distance may be a more appropriate for measuring accessible habitat for *M. daubentonii* and the NSL group. This supports previous studies highlighting the importance of linear landscape features [30], [46], [47], but this study is unique in demonstrating the functional importance of structural connectivity of tree cover for bat species in urban landscapes. As *M. daubentonii* has a similar wing aspect ratio and loading to the two *Pipistrellus* species we had expected models for *M. daubentonii* to include measures related to connectivity. It is possible that structural connectivity is relevant to this species, but that our landscape measures were insufficient to detect this relationship. However, given that our site data for this species is limited to activity within the first 1.5 hours after dusk and that this species is a late emerger, such interpretations should be treated with caution.

Whilst the concept is relatively straightforward, measuring functional connectivity in urban landscapes is challenging, particularly as the patch/matrix distinction is often unclear and actual movement paths are not easily observed. Previous studies have successfully employed expert judgement to estimate landscape resistance values for different urban matrix types [65]. Our approach is easily replicable and scalable, but relies on the accurate mapping of individual trees in three dimensions and on a consistent response by bats within a population to gaps in tree networks. For studies of highly mobile bird species, estimating the path of movement within the urban matrix has delivered improved models compared to more general landscape measures [11] and it is worth noting that for both *Pipistrellus* species studied, the only valid models we identified were those that included variables measured using a connectivity mask. At sites where the built land-cover of the surrounding landscape was over 40%, structural connectivity was critical for maintaining high levels of *P. pipistrellus* activity. Urban density dependent relationships with connectivity such as this have not been demonstrated before.

### Spatial scale

Gehrt and Chelsvig [28] and Lookingbill et al. [35] located bat survey sites within natural reserves along an urbanization gradient. However, the direct comparison of these studies is difficult as the spatial extent used to define urbanization and characterize the landscape differed between studies. We attempted to avoid such issues by measuring urban density at a wide range of spatial scales. This identified broad patterns, with edge specialists (*Pipistrellus* spp.) being sensitive to landscape composition even at small spatial scales (50–100 m). Their relatively fast and agile flight appears to allow them to utilise relatively small foraging areas, supported by a high wing aspect ratio, low wing loading and small size [27]. This may well explain their presence in densely built areas, as patch sizes tend to decrease with urbanization [6]. That *P. pygmaeus* responds to the landscape at radii of up to 1 km may reflect a need to travel further to access preferred feeding habitats [56] such as highly structured riparian vegetation [58]. The NSL aerial hawkers guild seeking un-built land-cover at larger scales (≥500 m) would be expected, given that their large size and high wing loading is suited to efficient flight over large open areas [27].

There are few studies that attempt to characterize the response of bats to urban landscapes at multiple radii that extend over a large spatial extent, although the response of groups such as birds has been explored [66]. Our data suggest that individual species may be sensitive to changes in landscape composition at multiple spatial scales. For example, evening models for *P. pipistrellus* include connected garden area within a radius of 500 m and connected tree cover within 150 m. We speculate that the 500 m radius may indicate the “roost catchment” of the pond i.e. that bats using the pond, tend to roost in houses within 500 m. The area within 150 m of a pond may be relevant to the quality and accessibility of the local feeding area, with ponds surrounded by a high density of tree networks being particularly desirable. These results corroborate other studies that conclude multiple spatial scales may be relevant to bats [35] and urban mammals [67].

### Conclusions

We have demonstrated that the density of landscape urbanization, its composition and configuration are important to the urban bat community. These relationships are scale dependent and species-specific. The broadly negative associations with urbanization for all species imply that a transition to more compact urban forms that reduce greenspace and habitat would inevitably impact the species richness of the urban bat community. This work informs the continuing debate about the sustainability of this approach to development [68]. The presence of thresholds for ecological function raises the possibility that development densities could be specified with ecological thresholds in mind, and that tipping points should be explored in more detail for other taxa. The importance of connectivity for the *Pipistrellus* species suggests that some ecological function could be retained even within high-density developments and that protected tree networks may deliver some spatial resilience [69] to the impacts of increased urban densities. It remains to be seen whether tree networks play a similar role for other organisms (but cf. [11]). Our data on ecologically important land-covers, land-uses and spatial scales should support urban planners and managers in making spatially explicit decisions about urban conservation [67]. We recommend that a multi-scale approach to planning and management be adopted, whilst recognising that this may be challenging given the typical spatial scales of urban land ownership [70], [71] and decision-making [72]. In particular, we suggest that when creating new urban bat habitats, consideration is given to ensuring that they remain functionally connected and therefore available to at least part of the urban bat community into the future.

## Materials and Methods

### Ethics Statement

The landowners gave permission for access to the sites. All Bat species are protected in the UK and licenses are needed if they are handled, mist-netted, or disturbed in their roosts. As our sampling involved only monitoring at foraging sites there were no licensing issues.

### Study Area and site selection

The West Midlands metropolitan county (population ∼2.3 million) is a highly urbanized region of the United Kingdom (UK), covering 902 km^2^. As a centre of the industrial revolution it has undergone multiple cycles of development and distinct zones can be identified representing pre, wartime and post-war regeneration. The study area includes several urban centres (Fig. 5) with high levels of sealed land-cover, canals, railways, residential areas of varying housing density, industrial zones, parks, nature reserves and agricultural land on the urban fringe. Existing survey records for the study area indicate that several species of bat were present, including: *Pipistrellus pipistrellus* (Schreber 1774), *Pipistrellus pygmaeus* (Leach 1825), *Myotis daubentonii* (Kuhl, 1817), *Eptesicus serotinus* (Schreber, 1774), *Nyctalus leisleri* (Kuhl, 1817) and *Nyctalus noctula* (Schreber, 1774). These species vary considerably in their roosting, commuting and feeding behaviour (Table S1).

In order to stratify the survey sites along an urbanization gradient we first classified the landscape using land-use and land-cover data from OS Mastermap (OSM) [73], which is a high-resolution parcel based GIS dataset (Table S1). OSM polygon data were converted into a 2 m pixel resolution raster and displayed in a GIS (ArcGIS 9.2, ESRI Redlands, USA). A grid of 1 km^2^ cells was used to extract raster summaries using Hawth's Analysis Tools [74], as this is close to the average minimum foraging areas of both *P. pipistrellus* and *P. pygmaeus* [75], which are the smallest foraging areas for the species we expected to encounter (Table S2). Five land classes were identified using a cluster analysis of landscape variable percentages (Table S1) in SPSS 18.0 [c.f. 76] and excluding squares with greater than 30% water cover or 80% tree cover. These represented a gradient from Rural (R), Light Suburban (LS), Suburban (S), Dense Suburban (DS) and Dense Urban (DU) land classes (Figs. 5&6). In order to reduce the potential for variations in local habitat composition to obscure the effect of landscape context [26], [77], survey sites were restricted to small (515–2146 m^2^) unlit ponds with at least 30% riparian edge tree cover. This choice was a reflection of the attractiveness of aquatic, riparian and woodland edge habitats for foraging to all the species we expected to record [78] and the need to identify a foraging habitat patch that would be present in all land classes. Candidate ponds were assigned to one of the five land classes based on the land-cover and land-use percentages for a 1 km^2^ circle surrounding each pond (Fig. 6) and six survey sites were then selected from each land class. All ponds were separated by at least one kilometre (pond centre to pond centre).

### Bat sampling methods

Ponds were surveyed for bat activity fortnightly between May and August 2009. We avoided nights where strong rainfall or wind were predicted and surveyed several sample points within each site [79]. A variety of techniques have previously been applied to compare bat species presence and activity between sites, with detectors generally regarded as superior [54]. We used a combination of walking transects [32], [51] and fixed point detector surveys [33], which allowed multiple microhabitats habitats to be surveyed and activity to be recorded from dusk to dawn.

Evening walking surveys were undertaken for a period of 1.5 hours following sunset using a Pettersson D240x ultrasound bat detector (Pettersson Electronic, Sweden), in heterodyne mode, alternating between 20 and 50 kHz. Sample calls of 3.4 seconds were recorded in time expansion mode and transferred to a Sony MZ_NH6000 Minidisk recorder (Sony, Japan). Walking routes circled each pond at varying distances (0- 50 m from edge), with the purpose of detecting and observing bats that were active in the close vicinity, as well as directly over the pond. Fixed point surveys were initiated at dusk and terminated at dawn, using an AnaBat SD1 frequency division bat detector (Titley Scientific, Australia) installed at the edge of the pond at a height of 1 m, using an acoustic reflector [80]. Tests confirmed that bats active within at least 15 m (horizontal distance) and up to ∼10 m above ground level were detectable, with bats calling at low frequencies (20–30 kHz) recorded at an unknown but greater distance.

### Call analysis

Bat calls were identified to species level where possible, using parameters given in Russ [78]. Where species identification was not possible in the field, bat calls detected on walking surveys were recorded and analyzed using BatSound 3.31 (Pettersson Electronic, Sweden). Calls recorded using fixed point Anabat detectors were processed automatically using filters within AnalookW [81]. Although *Myotis* sp. calls were identified at several sites the call quality was highly variable. Subsequent tests confirmed that using a reflector on the Anabat dramatically reduced the detectable range for this group, so *Myotis* calls from the fixed detector were excluded from analysis. Species or guild specific call filters were developed and their results compared to a 10% sample of the call dataset to estimate the percentage of bat calls incorrectly rejected by filters, calls allocated to the incorrect species/group, and files incorrectly identified as a bat call. Considerable caution was applied, preferring filters that discarded a greater percentage of calls. As considerable overlap in call parameters has been reported for *Nyctalus noctula*, *Eptesicus serotinus*, *Nyctalus leisleri*, and these species were rarely observed in flight (which would aid identification), we processed these (NSL) calls as a single functional group of large, early emerging bats with similar foraging behaviours (Table S2).

### Landscape and connectivity environmental variables

Using the GIS we selected a range of variables that related to roosting, commuting and feeding resources or that could be used as broad measures of urbanization (Table 2). Summaries of the area of built (sealed manmade) or natural (vegetated) surface cover (derived from the OSM landscape parcels) and the (remotely sensed) vegetation layer provided broad indications of urbanization density. Whilst the parcel based mapping was useful for estimating dominant land-cover, parcel types such as gardens were excluded as they contained varying levels of built and semi-natural land-cover. The remotely sensed vegetation layer was therefore used to gain a better reflection of vegetation cover. For the species we encountered, roost sites are likely to be located either in buildings or trees. Buildings provide a variety of roost opportunities due to their varied age, materials, architectural style and degree of maintenance. In addition to buildings, we included gardens from the OSM as an indirect measure of residential building availability, which we hypothesized might offer an enhanced roosting resource compared to commercial or industrial structures. Tree cover with a minimum height of either 15 m or 20 m was also included as a variable, as this was expected to indicate roost potential in mature woodland.

Several bat species are reported to fly along tree-lines when commuting and feeding [30], [78]. We estimated suitable commuting habitats by identifying areas of the landscape where vegetation was greater than 4, 6, 10 or 15 m high, which correspond to the range of typical flight heights for these species (Table S2). In addition, the raster representing vegetation greater than 4 m high was converted to a polygon feature class and buffered by a distance of 20 m. This resulted in a layer representing tree networks separated by gaps of no more than 40 m, which was used as a measure of structural connectivity and a proxy for functional connectivity. Although gaps of this size are not likely to be problematic for most species when commuting within rural landscapes [30], we hypothesized that with increasing urbanization, an increase in artificial lighting could be sufficient to deter bats from crossing such gaps [82]. Whilst we were unable to access spatial lighting datasets, we used a road dataset derived from the OSM as an indirect indicator of lighting and traffic disturbance. Insect feeding potential was represented by OSM derived polygons depicting water bodies (canals, streams and still waters), natural land-cover (predominantly vegetated) and supplemented with a high-resolution remotely sensed vegetation layer. Finally, the perimeter length of tree patches within tree networks connected to each pond was estimated, as many species are known to feed along woodland edges [47], [78].

Two approaches were taken to extract landscape variable summary data for the landscape surrounding each survey site. Firstly, using the GIS we created multiple concentric circular buffers around the ponds and extracted complete summaries of the underlying landscape data (Fig. 2A). Such a multi-scale approach is increasingly common [67], [83], although we used a particularly large number of radial extents (14, between 50 m and 4 km) in an attempt to accurately identify the spatial scales of relevance for each species. This approach assumes that all of the landscape is potentially available to the species concerned. Our second approach was to restrict the landscape analysis to the parts of the landscape adjacent to tree-lines. As both *P. pipistrellus* and *M. daubentonii* activity has been reported to occur predominantly within ∼50 m of water and woodland edges [31], [47], we buffered the tree networks connected to each pond by 50 m, creating a connectivity mask. Landscape variable summaries were again extracted at multiple radii around each pond centre, but this time the available landscape data were limited to the areas intersected by the connectivity mask (Fig. 2B).

### Data analysis

The measure of bat activity used for each site was the total number of minutes in which a call was recorded, for each species or functional guild (hereafter termed active minutes). We make no assumptions that this is a measure of individual bat abundance. Variation in bat activity between land classes was analyzed in SPSS using either a one-way ANOVA or a Kruskal-Wallace test if variances were heterogeneous. Tukey or Nemenyi post hoc tests were used to identify which classes differed in activity [84].

The relationships between the environmental measures and bat activity were modelled using a combination of Brodgar v2.6.4 (Highland Statistics, Newburgh, UK) and R (version 2.11.1) [85] using the mgcv and nlme libraries. We used up to three response variables per species (or guild): (i) the total minutes of bat activity recorded by the fixed detector for the first hour and a half following dusk (evening) (ii) total bat minutes recorded by the fixed detector from dusk to dawn (full-night) and (iii) presence using either the fixed detector or walking transect surveys (when bat activity data were either unavailable or did not produce valid models).

Data exploration was undertaken prior to statistical analyses, and as a result, we included an additional nominal explanatory variable differentiating between sites in highly urban (Dense Urban and Dense Suburban) and less urban land classes (Suburban, Light Suburban and Rural). This was used as a conditional variable in the fixed element of the models for some species (e.g. Table 1).

Initially we developed species or guild specific models independently for each spatial scale (50, 100, 150, 200, 350, 500, 750, 1000, 1250, 1500, 1750, 2000, 3000 and 4000 m). First, we used co-plots to inspect co-variation between explanatory variables at each scale and where variables had high correlations (>0.5) one of the pair was removed. We then assessed these against the response variables, removing explanatory variables with correlation scores of <0.3. This reduced the potential pool of explanatory variables considerably. None of the explanatory variables at higher spatial scales (>1000 m) showed significant relationships with any of our response variables. This left a pool of seven spatial variables at eight spatial scales (56 in total). We entered these into all the models and used Akaike Information Criterion (AIC) to identify the most parsimonious model for each species or guild, ensuring that model variables had variation inflation scores (VIFs) of <3 [86]. Where initial co-plots suggested linear relationships between the response and explanatory variables we used generalised linear modelling (GLM) [87]. Non-linear relationships were analyzed using generalised additive modelling (GAM) [88]. The deviance/degrees of freedom ratio was used to assess possible over-dispersion in the models [86]. We used negative binomial distributions to account for over-dispersion (Ne) [89] and logistic regression with a binomial (Bi) distribution for presence data [87]. Finally, this process was repeated with the variables from the best models at each scale combined to derive multi-scale models, pooling site based, concentric and connected variables for each species. All Models were validated using graphical visualisation tools in Brodgar and R. We plotted the residuals against fixed values to assess model homogeneity, QQ-plots for normality and plotted residuals against environmental co-variables to test for independence [90]. Lastly, we used bubble plots in the gstat R library to examine each individual model for spatial autocorrelation [91]. Where patterns indicated no spatial patterning the models were accepted (e.g. Figure S2). After validation we were left with a pool of 51 models: *Pipistrellus pipistrellus* evening (9), all-night (8); *Pipistrellus pygmaeus* evening (9), all-night (12), *M. daubentonii* evening (5) and NSL guild evening (8). Of these, candidate models with AIC≤2 of the optimum model were retained [92] (Table 1).

## Supporting Information

Figure S1 **Residual bubble plot for NSL all-night Anabat data from logit binomial presence-absence data.** The plot shows clumping of similar size positive residuals in the middle of the plot, indicative of spatial structuring in the data. Negative residuals in black and positive residuals are grey. The size of the circles indicates the size of the residuals.
(TIF)

Figure S2 **Bubble plot for** ***P. pipistrellus*** **all-night Anabat residuals from a GAM of bat activity minutes.** The plot indicates no spatial structuring in the data. Negative residuals in black and positive residuals are grey. The size of the circles indicates the size of the residuals.
(TIF)

Table S1 **Mean area (m2) and standard deviation of Ordinance Survey (OS) land-cover type for each urban land class.**
(DOC)

Table S2 **Broad life history data for bat species recorded within the study area.**
(DOC)
