# Supplementary figures and images for: Habitat Composition and Connectivity Predicts Bat Presence and Activity at Foraging Sites in a Large UK Conurbation
Source: PLoS One. 2012 Mar 12;7(3):e33300. doi: 10.1371/journal.pone.0033300 (PMC3299780; doi:10.1371/journal.pone.0033300)

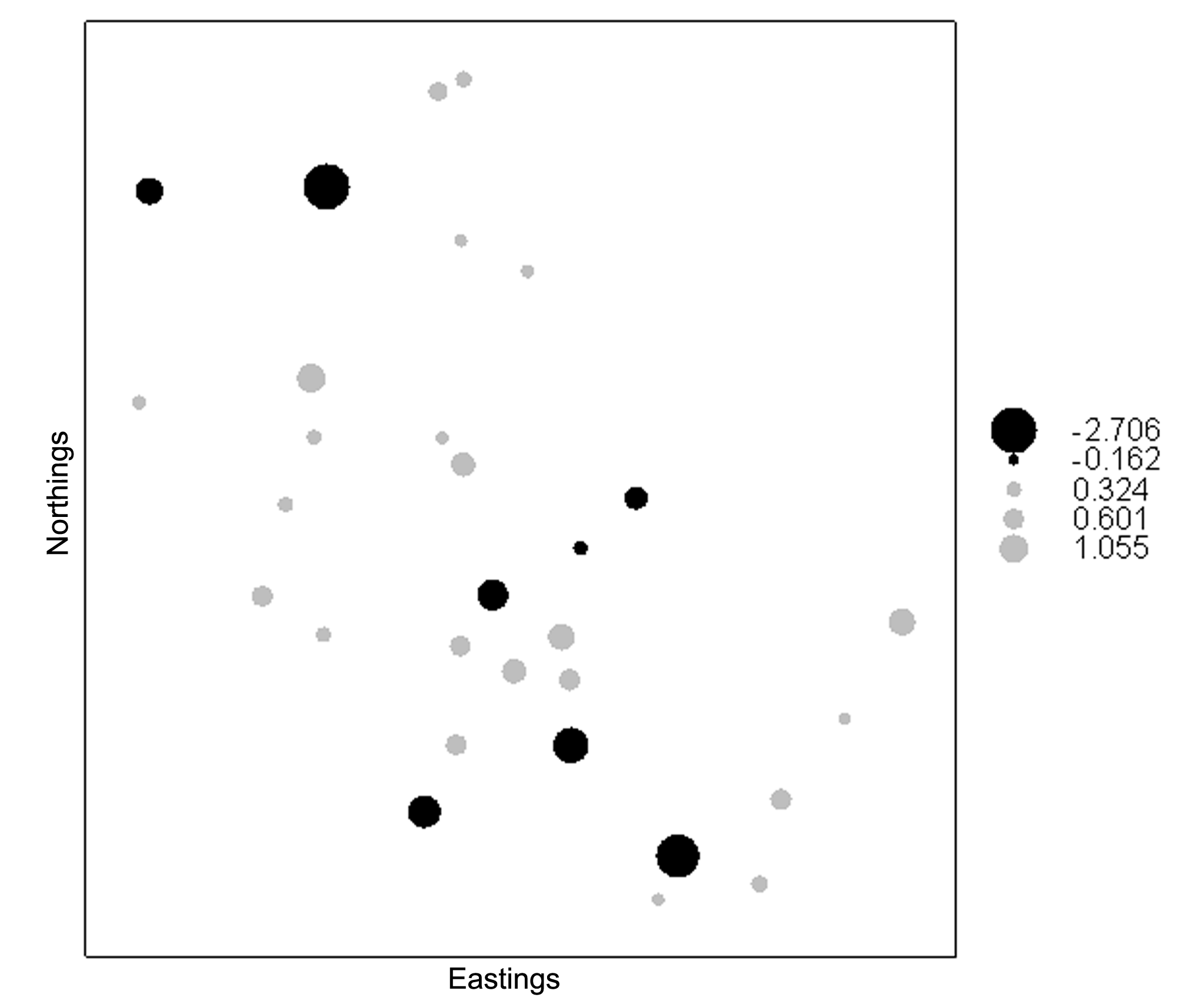

Supplement: Figure S1 — Residual bubble plot for NSL all-night Anabat data from logit binomial presence-absence data. The plot shows clumping of similar size positive residuals in the middle of the plot, indicative of spatial structuring in the data. Negative residuals in black and positive residuals are grey. The size of the circles indicates the size of the residuals. (TIF) [file pone.0033300.s001.tif]

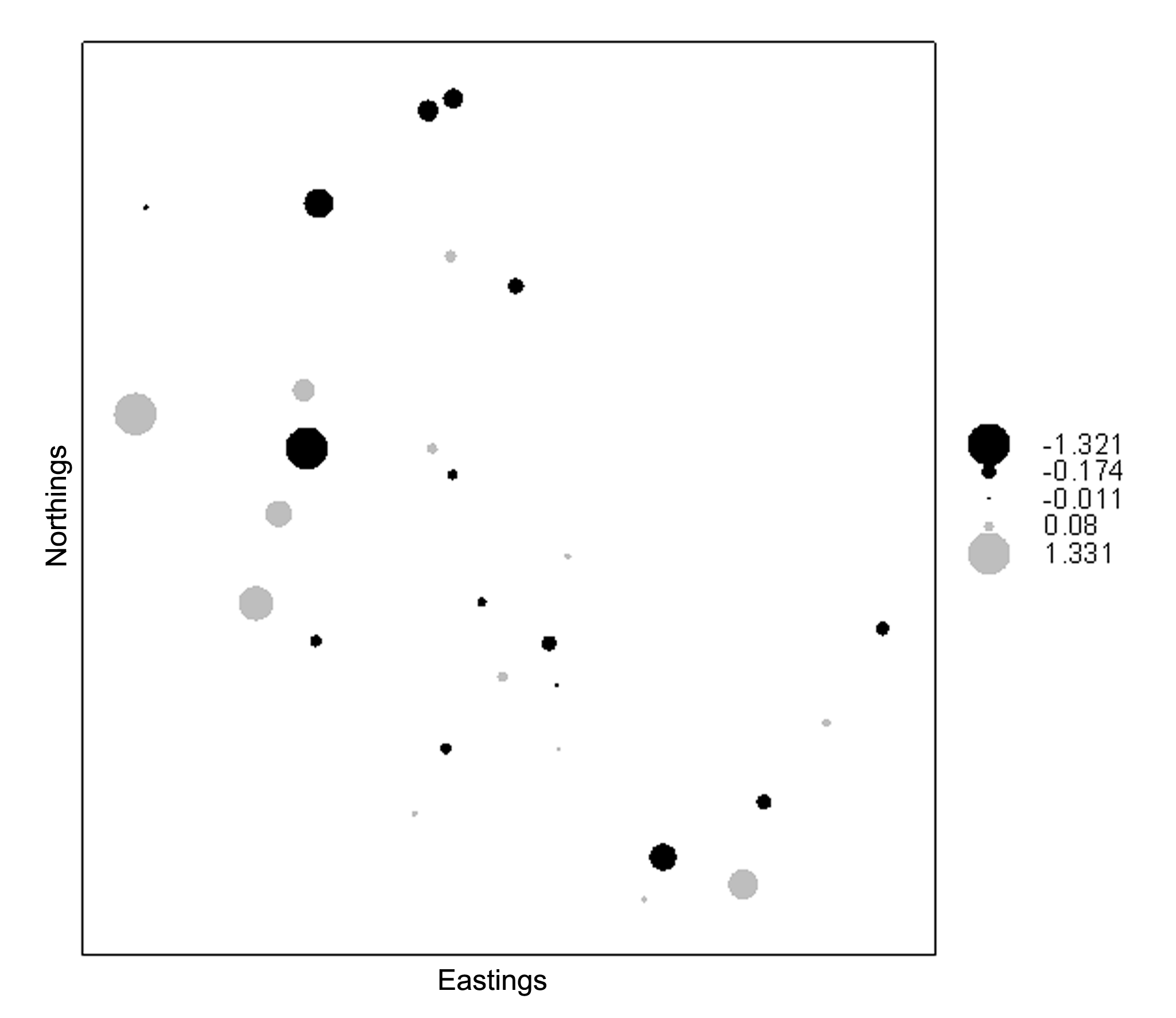

Supplement: Figure S2 — Bubble plot for P. pipistrellus all-night Anabat residuals from a GAM of bat activity minutes. The plot indicates no spatial structuring in the data. Negative residuals in black and positive residuals are grey. The size of the circles indicates the size of the residuals. (TIF) [file pone.0033300.s002.tif]
