# Supplementary material for: Habitat Composition and Connectivity Predicts Bat Presence and Activity at Foraging Sites in a Large UK Conurbation
Source: PLoS One. 2012 Mar 12;7(3):e33300. doi: 10.1371/journal.pone.0033300 (PMC3299780; doi:10.1371/journal.pone.0033300)
Supplement: Table S1 — Mean area (m2) and standard deviation of Ordinance Survey (OS) land-cover type for each urban land class. (DOC) [file pone.0033300.s003.doc]

**Table S1.** Mean area (m^2^) and standard deviation of Ordinance Survey (OS) land-cover type for each urban land class.

|  |  | **Built structures** | | **Gardens 0-400m^2^** | | **Gardens>400 m^2^** | | **Manmade (built)**  **Open Space** | | **Canals/rivers** | | **Stillwaters** | |
| --- | --- | --- | --- | --- | --- | --- | --- | --- | --- | --- | --- | --- | --- |
| **Class name** | **Class** | **Mean** | **SD** | **Mean** | **SD** | **Mean** | **SD** | **Mean** | **SD** | **Mean** | **SD** | **Mean** | **SD** |
| Rural | 1 | 5,227 | 4,726 | 6,007 | 9,076 | 17,552 | 12,281 | 14,370 | 9,107 | 709 | 1,161 | 1,313 | 1,407 |
| Light suburban | 2 | 14,266 | 11,282 | 18,680 | 20,991 | 37,781 | 33,742 | 24,876 | 15,240 | 2,011 | 1,837 | 7,659 | 9,107 |
| Suburban | 3 | 24,229 | 7,944 | 40,745 | 19,722 | 32,464 | 17,848 | 37,274 | 15,173 | 1,246 | 1,517 | 1,164 | 1,874 |
| Dense suburban | 4 | 37,502 | 8,858 | 76,756 | 18,492 | 17,820 | 10,781 | 39,164 | 9,631 | 562 | 942 | 370 | 961 |
| Dense urban | 5 | 51,307 | 15,910 | 30,202 | 17,521 | 9,573 | 9,035 | 65,137 | 17,939 | 3,440 | 2,240 | 597 | 1,338 |

|  |  | **Motorways** | | **A_roads** | | **B_roads** | | **Minor roads** | | **Natural (vegetated) open spaces** | | **Railways** | | **Trees** | |
| --- | --- | --- | --- | --- | --- | --- | --- | --- | --- | --- | --- | --- | --- | --- | --- |
| **Class name** | **Class** | **Mean** | **SD** | **Mean** | **SD** | **Mean** | **SD** | **Mean** | **SD** | **Mean** | **SD** | **Mean** | **SD** | **Mean** | **SD** |
| Rural | 1 | 233 | 991 | 774 | 1,565 | 544 | 941 | 3,413 | 2,372 | 192,492 | 26,198 | 258 | 744 | 1,484 | 2,322 |
| Light suburban | 2 | 4,775 | 5,167 | 1,740 | 2,141 | 353 | 777 | 6,743 | 4,729 | 123,097 | 47,319 | 783 | 1,435 | 5,169 | 7,911 |
| Suburban | 3 | 52 | 435 | 1,653 | 1,919 | 753 | 1,140 | 11,794 | 3,641 | 94,461 | 31,104 | 789 | 1,664 | 941 | 2,008 |
| Dense suburban | 4 | 122 | 804 | 2,537 | 2,586 | 1,191 | 1,550 | 18,301 | 3,471 | 53,300 | 18,816 | 766 | 1,483 | 70 | 429 |
| Dense urban | 5 | 973 | 2,633 | 4,809 | 3,488 | 2,053 | 2,416 | 15,567 | 4,973 | 59,567 | 19,678 | 4,982 | 6,354 | 93 | 545 |

Urban land classes were derived using a cluster analysis of OS land-cover data for 902 individual km squares
