# Supplementary material for: Habitat Composition and Connectivity Predicts Bat Presence and Activity at Foraging Sites in a Large UK Conurbation
Source: PLoS One. 2012 Mar 12;7(3):e33300. doi: 10.1371/journal.pone.0033300 (PMC3299780; doi:10.1371/journal.pone.0033300)
Supplement: Table S2 — Broad life history data for bat species recorded within the study area. (DOC) [file pone.0033300.s004.doc]

**Table S2.** Broad life history data for bat species recorded within the study area.

| **Species** | **Summer roost preference** | **Emergence** | **Feeding preference** | **Commuting /flight preference** | **Typical maximum stray distance (km) from tree line** | **Av dist (km) from maternity roost to feeding (pre/during lactation).** | **Max dist (km) from maternity roost to feeding (pre/during lactation)** | **Av max distance (km) to roost per feeding bout**  **lactating/not** | **Av MCP (km^2^)** | **Sources** |
| --- | --- | --- | --- | --- | --- | --- | --- | --- | --- | --- |
| *Pipistrellus*  *pipistrellus (45kHz)* | Built structures – within gaps and under cladding  Preference for pre 1945 and damaged buildings | Average | Edge  Half open areas  Riparian vegetation and woodland edge | Follows structures  Not always shortest route  5-10 m above ground | Will cross open fields of 150-200  Activity highest within 50m of river/tree edge | 1.8 (n=8)  1.8/1.3 | 2.0  5.1/3.7 | 1.5 | 0.92 | Simon et al. 2004  Russ 1999  Downs & Racey 2006  Verboom & Huitema 1997  Davidson-Watts & Jones 2006 |
| *Pipistrellus pygmaeus*  *(55kHz)* | Predominantly built structures | Average | Edge  Half open areas  Strong riparian preference but also woodland edge | Follows structures  5-10 m above ground | Activity predominantly within 50m of river | 1.8/1.3 | 5.1/3.7 | 1.7 | 1.10 | Russ 1999  Simon et al. 2004  Downs & Racey 2006  Racey & swift 1985  Davidson-Watts and Jones 2006 |
| *Myotis daubentonii* | Structures such as stone bridges  Tree hollows | Late | Close to water  Closed canopy  Woodland edge | Follows structures | Activity predominantly within 20m of river | 2.4 (n=14)  2.3 | 3.8  6.3 |  |  | Lucan & Radil 2010  Russ 1999  Downs & Racey 2006 Kapfer et al. 2008  Dietz et al. 2006 |
| *Eptesicus serotinus* | Built structures – within gaps and under cladding | Early | Open/Edge  Parks and gardens of populated areas Pasture  Woodland edge | Follows structures  Shows a preference for areas with linear features  1-15m | Will cross open fields of 150m but activity reduces with distance woodland edge | 1.3 | 5.7  7.4 |  | 7.5 | Simon et al. 2004  Russ 1999  Verboom & Huitema 1997  Robinson & Stebbings 1997 |
| *Nyctalus leisleri* | Oak and ash trees that are larger than others locally available. 15-42m | Early | Open  Wetlands  Parkland  Woodland edge | 10 m, up to ~ 70 m |  |  | 13.4 | ~ 4 to ~7  4.2 | 7.4 | Russ 1999  Ruczyn & Bogdanowicz 2008  Sheil et al. 1999  Waters et al. 2006 |
| *Nyctalus noctula* | Woodpecker tree hollows. Beech and  Oak trees, larger than others locally available. 18_44m | Early | Open  Woodland  Pastures  Lakes | >10m |  |  |  | 4.2  3.8/4.7 (Median) | 8.2 | Boonman 2000  Racey 1991  Ruczyn & Bogdanowicz 2008  Russ 1999  Mackie & Racey 2007 |

A selection of information relating to land-cover preferences and flight behaviour of bats recorded in this study are presented, along with references for source data.
